# Spectral Preferences of *Encarsia formosa:* Unravelling Attraction to LED Monitoring Traps

**DOI:** 10.3390/insects17030246

**Published:** 2026-02-26

**Authors:** Emeka Emmanuel Ekejiuba, Rainer Meyhöfer

**Affiliations:** Section Phytomedicine, Applied Entomology, Institute of Horticultural Production Systems, Leibniz Universität Hannover, Herrenhäuser Str. 2, D-30419 Hannover, Germany; emmaemeka141@gmail.com

**Keywords:** natural enemy, visual behavior, biological control, whitefly, *Trialeurodes vaporariorum*, greenhouse, tomato, *Encarsia formosa*

## Abstract

LED-enhanced sticky traps improve monitoring of greenhouse whitefly, *Trialeurodes vaporariorum* Westwood (Hemiptera: Aleyrodidae), but their effects on its parasitoid, *Encarsia formosa* Gahan (Hymenoptera: Aphelinidae), are unclear, which may compromise biological control. Methods: We quantified *E. formosa* visual responses in climate-chamber multiple-choice arenas using six LED colors at equal photon flux (8.1 μmol m^−2^ s^−1^) and in greenhouse choice/no-choice assays comparing a standard yellow sticky trap with a green LED-enhanced yellow trap, with and without host-infested tomato leaves. We further tested modified LED traps (green LEDs with white or green backgrounds) and assessed intensity-dependent responses (18.0–25.6 μmol m^−2^ s^−1^). Results: *E. formosa* showed the highest attraction to green LEDs (peak ≈ 521–524 nm) and a significantly lower response to other colors. In greenhouse assays, *E. formosa* preferred the standard yellow sticky trap over the LED-enhanced yellow trap; in no-choice tests, only 9% were recaptured on the LED-enhanced yellow trap, both without and with hosts. Modified traps with white or green backgrounds substantially increased *E. formosa* recapture (≈54% higher than the yellow-background LED trap). *Encarsia formosa* attraction to the white-background LED trap declined with increasing intensity (61% at 18.0 to 4% at 25.6 μmol m^−2^ s^−1^), whereas whitefly captures were stable to slightly higher. Conclusions: The standard LED-enhanced yellow trap is compatible with *E. formosa* releases and does not disrupt biocontrol. Modified LED traps show promise for simultaneous monitoring of *E. formosa* and whiteflies, warranting validation under commercial conditions.

## Simple Summary

Greenhouse whitefly is a serious pest of tomatoes and other crops. Growers often use yellow sticky cards to track its numbers, and newer traps with built-in light sources have made detection more reliable. However, there is a concern that these brighter traps might also lure in the whitefly’s natural enemy, a tiny parasitic wasp called *Encarsia formosa* Gahan (Hymenoptera: Aphelinidae) which helps keep whiteflies under control. We tested how this wasp responds to different trap colors and to lighted traps in controlled rooms and greenhouses, with and without whitefly-infested leaves. The wasp was most attracted to green light but, importantly, it was rarely caught by the standard whitefly monitoring trap that combines a yellow card with green light. This means the commonly used lighted trap can be used alongside the wasp without disrupting biological control. We also built modified traps with white or green backgrounds that increased the captures of both the wasp and whiteflies at lower light levels, suggesting that they could monitor both beneficials and pests at the same time. These findings support more precise, pesticide-saving pest management, helping growers protect crops while reducing environmental impacts.

## 1. Introduction

Insects perceive and use color information in many behavioral contexts, from navigation to host and mate location, yet their visual systems differ markedly across taxa in receptor complements, spectral sensitivity, and chromatic processing [1,2,3]. These differences are exploited in integrated pest management (IPM) through visually based monitoring, most commonly with colored sticky cards. Yellow sticky cards, in particular, have long been used to detect and track the population dynamics of greenhouse pests such as the greenhouse whitefly, *Trialeurodes vaporariorum* [4,5,6,7], while blue cards target several thrips species [8]. However, the performance of passive color traps depends on ambient illumination and the spectral reflectance of the colored card, which can limit sensitivity and specificity [9]. In contrast, light-emitting diodes (LEDs) offer narrowband spectra and stable, controllable intensities that can be tuned to insect photoreceptors, and LED-enhanced traps have improved the capture of several greenhouse pests, including whiteflies and thrips [9,10,11,12]. For *T. vaporariorum*, green LEDs near 520 nm are particularly effective [9,11,13]. A critical, unresolved question is whether such LED-enhanced traps inadvertently attract natural enemies and thereby compromise biological control. *Encarsia formosa*, a key parasitoid of *T. vaporariorum* and widely used in commercial biocontrol [14,15], is known to be caught on yellow cards [6], but its spectral preferences and responses to LED-enhanced traps are poorly characterized [16]. Previous studies diverge: some report low by-catch of parasitoids on LED-enhanced traps [17,18], others did not assess natural enemies [9,19], while field observations suggest that LED-enhanced sticky traps may be less attractive to *E. formosa* than standard yellow cards [16]. Moreover, chromatic opponency—documented in whiteflies as a blue–green mechanism that modulates responses to green stimuli [11,20]—could further complicate predictions for parasitoid behavior, particularly when LED emissions are combined with broad-spectrum yellow backgrounds. Additionally, trap catches depend most likely on the species-specific daily foraging activities of the parasitoid in the crop stand [21]. Here, we address these gaps by (i) quantifying the spectral preferences of *E. formosa* across six LED colors at equal photon flux; (ii) testing the parasitoid’s response to a standard yellow sticky card versus a green LED-enhanced yellow monitoring trap, with and without host-infested tomato leaves; (iii) evaluating modified LED trap variants with white or green backgrounds; and (iv) examining intensity-dependent responses relevant to practical deployment. We hypothesize that the yellow color is less attractive for the parasitoid [6,22], at least in the presence of suitable host nymphs, which also contributes to low captures on the LED-enhanced yellow whitefly trap [16], and therefore guarantees compatibility with biocontrol releases.

## 2. Materials and Methods

### 2.1. Plant and Insect Rearing

All whiteflies used in the experiments were reared on Brioso F1 hybrid tomato plants (*Solanum lycopersicum*) sourced from Rijk Zwaan (De Lier, The Netherlands). Seeds were sown in nursery pots with peat substrate and grown for 32 days under standard conditions in the greenhouse, and were then transferred to a climate chamber with flight cages (0.97 m × 0.63 m × 0.59 m) maintained at 22 °C and 50–60% relative humidity. Each of the four net-covered cages (80 MESH) contained three plants. Fifty adult whiteflies from an established colony were introduced to the plants using glass vials. Plants were watered every second day until a sufficient population developed for experiments.

*Encarsia formosa* used in all experiments was obtained from Katz Biotech AG (Baruth/Mark, Germany). The parasitoids were supplied as parasitized (black) whitefly nymphs embedded in circular patches on hanging paper cards. Cards were placed in a small plastic rearing box (15 × 8 × 11 cm) covered with *Encarsia* safe mesh. A piece of cotton wool soaked with 2.4 g of water was provided as a water source for emerging adults. Rearing conditions were 26 ± 1 °C and a photoperiod of 18 h light:6 h dark. All *E. formosa* used in experiments were starved for a minimum of 15 h to standardize hunger levels. Immediately before each experiment, newly emerged adults were collected using a fine insect brush.

### 2.2. Investigating Spectral Preferences of Encarsia formosa in Multiple Choice Arenas

Assays were conducted in a climate chamber at 25 ± 1 °C with an 18 h light:6 h dark cycle. We used the LED choice arena of Stukenberg et al. [11], but positioned the LED traps additionally in the upper part of the cage rather than at the bottom. Two cages (0.8 m × 1 m × 1 m) were used simultaneously. Six high-power LEDs (UV, blue, cyan, green, yellow, and red; Table 1) were installed, with the insect release point 0.7 m from the traps. Each LED trap was a cube-shaped box made of PVC boards with an opening at the back for the LEDs, which were connected to DC via AC–DC converters (Mini Jolly, TCI, Saronno, Italy). The front was covered with a slide-in acrylic glass (PLEXIGLAS LED 0M200 SC, Evonik Industries AG, Essen, Germany) serving as a light-scattering screen. The interior was lined with mirror foil (PEARL GmbH) to ensure even light distribution. The acrylic surface was covered with cellophane gift-wrapping foil manually coated with insect glue (Temmen GmbH, Hattersheim, Germany). The sticky film was secured to the cube-shaped box with a rubber band. Each LED trap measured 0.10 × 0.10 × 0.13 m and was mounted on a 0.62 m high platform.

Following Stukenberg et al. [11], the intensity of each LED trap (except UV) was measured and adjusted to equal photon flux (μmol m^−2^ s^−1^) using a LI-250 light meter with a LI-190 quantum sensor (LI-COR Biosciences GmbH, Bad Homburg, Germany) and ProfiLab-Expert 4.0 (ABACOM, Ganderkesee, Germany) to operate and automate illumination modes. UV intensity was measured with a UV-A sensor type 2.5 (Ahlborn GmbH, Bodenwerder, Germany), and photon flux density was calculated from W m^−2^ using the spectrum, Planck’s constant, and Avogadro’s number. All traps were set to 8.1 μmol m^−2^ s^−1^. Emission spectra were recorded with an Avaspec 2048-2 spectrometer (Avantes, Apeldoorn, The Netherlands).

Newly emerged, unfed, unmated *E. formosa* (approximately 24 h old) were used in the choice arenas. Forty wasps were released from Petri dishes (0.02 m high, 0.10 m diameter) placed on 0.04 m high platforms at the designated release points. The experiment was replicated 20 times, using cages as the experimental unit (two replicates per day over 10 non-consecutive days). Each trial lasted 24 h, after which the cages were cleaned and the trapped insects counted. After each replicate, fresh cellophane film was mounted and coated with glue. Trap positions were randomized after each trial.

### 2.3. Response of Encarsia formosa to LED-Enhanced and Standard Whitefly Monitoring Traps

The LED-enhanced yellow trap combined a non-sticky yellow card (IVOG biotechnical systems GmbH, Kissing, Germany) with green high-power LEDs (NCSG219BT-V1 SMD, Nichia Corporation, Anan, Japan) [11,19]. Briefly, the device consisted of a U-shaped aluminum frame holding a layer of white PVC plate, yellow card, transparent acrylic for LED edge lighting (PLEXIGLAS LED 0E010 SM, Evonik Industries AG), and a transparent acrylic cover (PLEXIGLAS XT clear 0A000 GT, Evonik Industries AG) on top. Eight green LED chips (521 nm) were arranged two per side into the frame, illuminating the acrylic plate for edge lighting. The traps were operated with multi-stage constant-current drivers (LCM-40, MEAN WELL, New Taipei, Taiwan) at 80 V and 0.5 A. The front surface of the traps was also covered with cellophane gift-wrapping foil and coated with insect glue (Temmen GmbH, Hattersheim, Germany). Standard yellow cards were cut to the same square size as the LED traps and were also covered with the coated cellophane film. All experiments were conducted in a greenhouse using flight cages (1.31 m × 1.71 m × 0.87 m) placed on 0.73 m high tables. The cages were separated with black polyethylene foils to prevent interference among light traps. Ambient temperature and light intensity were recorded every 30 min with data loggers; mean values were 27 °C and 2109 lx.

Two sets of experiments, i.e., choice and non-choice tests, were conducted. A choice test without host plants investigated the preference of the parasitoid for LED-enhanced yellow traps compared to standard yellow sticky traps. Therefore, traps were positioned at a distance of 0.24 m from each other, while 10 starved adult *Encarsia formosa* (unfed from emergence) were released at a distance of 0.77 m from a Petri dish (0.02 m × 0.07 m diameter). Experiments were conducted in the greenhouse flight cages on a daily basis, with three runs per day scheduled at 8:00–11:00 in the morning, 12:00–15:00 in the afternoon, and 15:30–19:00 in the evening. After each run, the trapped *Encarsia* were counted. Treatments were completely randomized and replicated 24 times for subsequent statistical analysis.

In the no-choice tests, we investigated the attractiveness of LED-enhanced traps for *Encarsia formosa* in the absence and presence of whitefly hosts. In the absence of host nymphs, 10 starved adult *Encarsia formosa* were released from a Petri dish at a distance of 0.77 m from the trap. In the presence of hosts, 6 starved adult *Encarsia formosa* together with 6 adult whiteflies (positive control) were placed together into a Petri dish containing a tomato leaf infested with 30–50 whitefly nymphs as a resource for food and egg laying. Shortly after, open Petri dishes were placed at a distance of 0.77 m from the trap. The experiment was replicated 10 times and the duration of each experiment was 48 h, after which the *E. formosa* and whiteflies caught in the trap were counted, while parasitism was estimated 16–21 days later for subsequent statistical analysis.

### 2.4. Response of Encarsia formosa to Modified Whitefly LED Monitoring Traps

To assess responses to modified traps, we replaced the yellow background of the LED-enhanced trap with white or green background cards (IVOG biotechnical systems GmbH, Kissing, Germany). For reference, the modified trap with a green background had an intensity of 1.03 μmol m^−2^ s^−1^ at a peak wavelength of 524.03 nm, while the white background version had an intensity of 0.81 μmol m^−2^ s^−1^ at 524.03 nm. Experiments were conducted again in the greenhouse using the same flight cages (see above). In no-choice tests without hosts, the LED-enhanced trap (green or white background) was placed in the cage, and 20 *E. formosa* and 20 whiteflies (positive control) were released from a Petri dish at a distance of 0.77 m from the trap. Experiments were completely randomized, replicated 20 times, and conducted between 09:00 and 15:00. In no-choice tests with hosts, single tomato leaves (≤25 cm^2^) bearing 30–50 whitefly nymphs were placed in Petri dishes with water-soaked cotton around the petiole. Six female *E. formosa* and 6 adult whiteflies were released simultaneously on the leaf, which was positioned 0.77 m from the LED-enhanced trap (white background only). The experiment was replicated 10 times and lasted 48 h. After each trial, trapped insects were counted and parasitism was estimated as described above.

Finally, in no-choice assays, the response of both insects to varying LED intensity levels was investigated with the white-background card only. Trap LED intensity was varied by adjusting the current input (LCM-40, MEAN WELL) and measured at four points 0.17 m from the trap (release point) using a LI-250 light meter with an LI-190 quantum sensor. Intensities were 18.03, 22.20, 24.10, and 25.60 μmol m^−2^ s^−1^ at current settings 0, 2, 4, and 6, respectively. A single sticky LED trap was placed in a flight cage (see above) and 10 starved *E. formosa* (20 h old), together with 10 adult whiteflies starved for 3 h (positive control) were released from Petri dishes. Experiments were replicated three times with each of the four intensity settings, and captures were recorded for subsequent statistical analysis.

### 2.5. Statistical Analysis

Model selection was conducted prior to analysis using the hnp package in R (version 22.12.0). Data visualization used the ggplot() function in ggplot2 [23]. Percentage recapture for each species and trap was calculated as the count of individuals recaptured on a given trap divided by the total number recaptured across species, multiplied by 100. Data were analyzed using generalized linear models (GLMs) in R; ANOVA was performed within the GLM framework, assuming a Poisson distribution for quantitative data and a binomial distribution for proportions, respectively, while accounting for overdispersion. Odds ratios were estimated to obtain average proportions and 95% confidence intervals of insects trapped in each treatment. The response variable was the number of insects trapped; explanatory variables included trap type, experimental period, and cage, with interaction terms evaluated where appropriate. Tukey’s pairwise comparisons were conducted using the emmeans package (version 2.0.1) [24]; data were log-transformed where required. A *p*-value < 0.05 was considered statistically significant.

## 3. Results

### 3.1. Visual Preference of Encarsia formosa for Different LED Spectra

**Figure 1:**
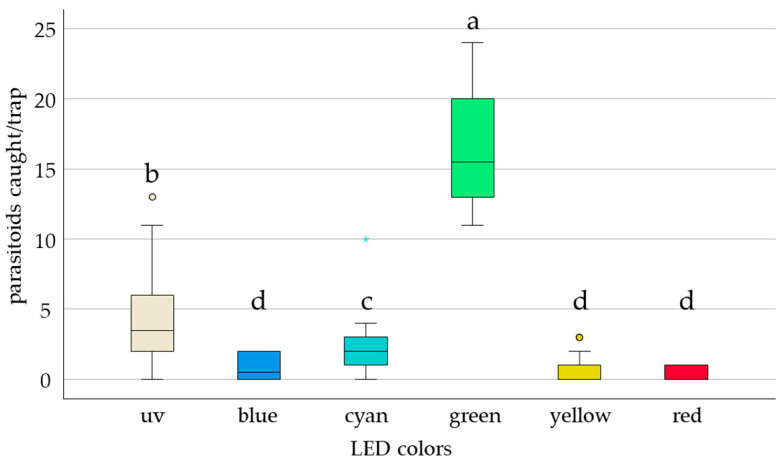
Box plot showing the median numbers of *E. formosa* caught in traps with different LED colors in a multiple-choice experiment after 24 h exposure time. In total, 40 *E. formosa* were released per replicate (n = 20). Significant differences (odds ratio) are represented by different letters (ANOVA, GLM, Logit-link, α ≤ 0.05, Tukey). Open circles and asterisks show outliers above 1.5 times the inter-quartile range.

Of all *E. formosa* released, 61% were recaptured on the six different LED traps (3.81 ± 0.79). LED color had a significant effect on the mean recapture rate (F = 94.7, df = 5, *p* = 0.001) (Figure 1). Specifically, the green LED yielded a significantly higher recapture rate than all other colors (67%, *p* = 0.0001). Recapture under UV also differed significantly from other colors (17%, *p* = 0.0026). However, there was no significant difference in recapture among the blue, red, cyan, and yellow LEDs (Figure 1).

### 3.2. Behavioral Response of Encarsia to Whitefly Monitoring Traps

**Figure 2:**
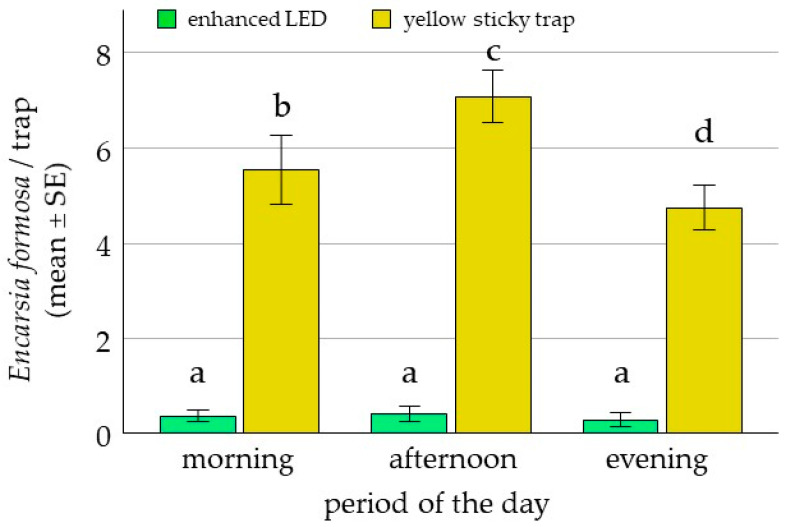
Mean number (±SE) of *E. formosa* trapped on LED-enhanced traps and standard yellow sticky traps at different times of day in choice tests: morning (8:00–11:00), afternoon (12:00–15:00), and evening (15:30–19:00). In total, 10 *E. formosa* were released per replicate (n = 24). Significant differences (odds ratio) are represented by different letters (ANOVA, GLM, Logit-link, α ≤ 0.05, Tukey).

**Figure 3:**
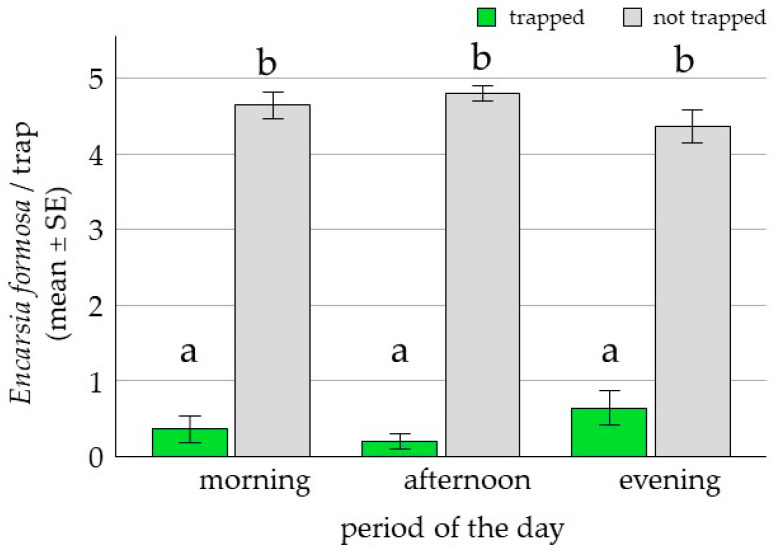
Mean number (±SE) of *E. formosa* trapped and not trapped on LED-enhanced traps at different times of day in no-choice tests: morning (8:00–11:00), afternoon (12:00–15:00), and evening (15:30–19:00). Number of *E. formosa* released per replicate = 6, n = 20 (GLM, binomial, Logit-link, α ≤ 0.05, Tukey). Significant differences (odds ratio) are represented by different letters.

**Figure 4:**
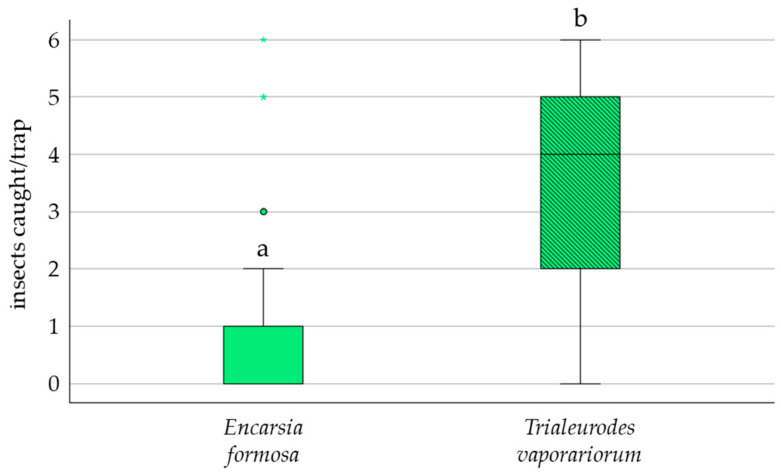
Box plot showing the median number of *E. formosa* and whiteflies trapped on LED-enhanced traps over a 48 h exposure time in a no-choice test (nymphs on tomato leaves present). Six *E. formosa* were released per replicate (n = 10). Significant differences (odds ratio) are represented by different letters (ANOVA, GLM, Logit-link, α ≤ 0.05, Tukey). Open circles and asterisks show outliers above 1.5 times the inter-quartile range.

In the choice tests, the standard yellow sticky trap captured a significantly higher proportion of *E. formosa* (94%; 2.69 ± 0.41, *p* = 0.016) than the LED-enhanced yellow trap (6%) (Figure 2). Responsiveness increased approximately fivefold from morning to afternoon; however, the interaction between trap type and time of day was not significant (*p* = 0.968). In no-choice tests, most *E. formosa* (91%) did not respond to the LED-enhanced yellow trap, and only 9% were recaptured (1.52 ± 0.58, *p* = 0.012) (Figure 3). While foraging on tomato leaves, only 9% of *E. formosa* were trapped on the LED-enhanced yellow trap (Figure 4). By contrast, there was a significant difference between the proportions of *E. formosa* and whiteflies trapped (P[*E. formosa* trapped]/P[whiteflies trapped] = 0.53 ± 0.11, *p* = 0.0001).

### 3.3. Behavioral Response to Modifications of the Whitefly LED Monitoring Trap at Different Intensities

**Figure 5:**
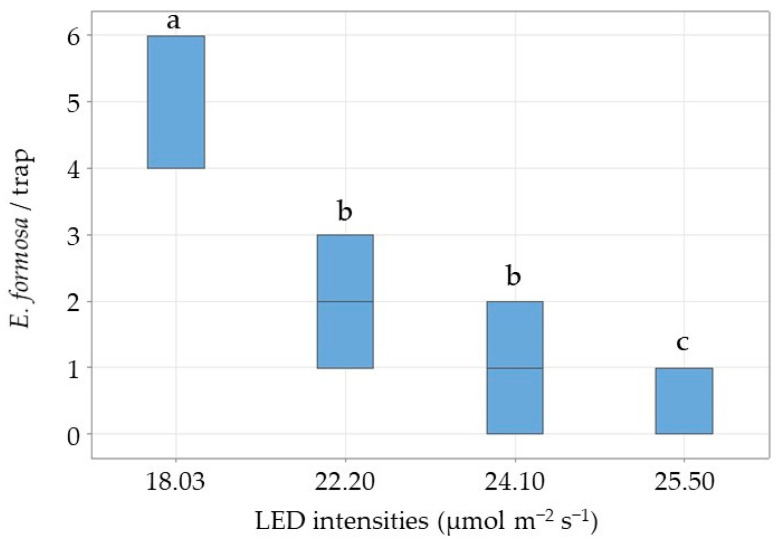
Box plot showing the median number of *E. formosa* per trap at four different green LED intensity levels and with a white background. In total, 10 *E. formosa* were released per replicate (n = 12). Significant differences (odds ratio) are represented by different letters (ANOVA, GLM, Logit-link, α ≤ 0.05, Tukey).

**Figure 6:**
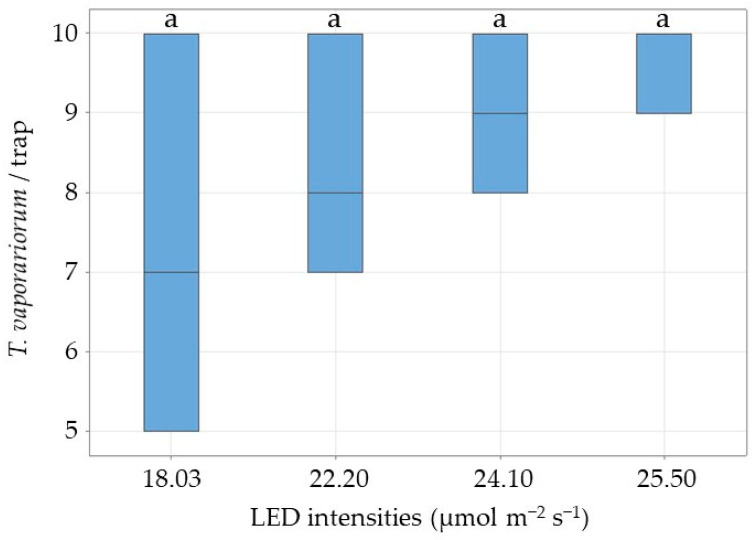
Box plot showing the median number of *T. vaporariorum* per trap at four different green LED intensity levels and with a white background. In total, 10 female whiteflies were released per replicate (n = 12). Significant differences (odds ratio) are represented by letters (ANOVA, GLM, Logit-link, α ≤ 0.05, Tukey).

*Encarsia formosa* responses to the modified trap with a white background decreased significantly with increasing green LED intensity, from 61% at 18.03 μmol m^−2^ s^−1^ to 4% at 25.60 μmol m^−2^ s^−1^ (2.41 ± 0.05, *p* = 0.005) (Figure 5). Whitefly response to the same trap increased as expected, from 22% at 18.03 μmol m−2 s−1 to 27% at 25.60 μmol m^−2^ s^−1^ (Figure 6). Across intensities, there was no significant difference in the mean recapture rate of T. vaporariorum (1.67 ± 0.21, *p* = 0.438) (Figure 6).

**Figure 7:**
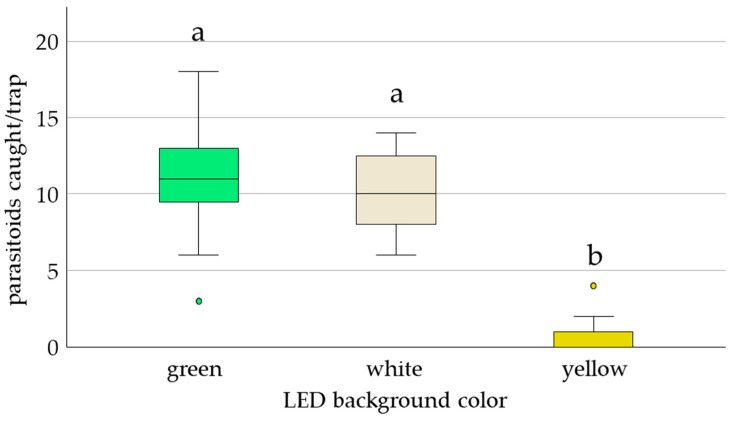
Box plot showing the median number of *E. formosa* caught on LED-enhanced monitoring traps (yellow background) and modified LED traps (white and green backgrounds) in no-choice situations. Trap spectral ranges: 524 nm for green and white background traps, and 525 nm for yellow background trap. In total, 20 *E. formosa* were released per replicate (n = 20). Significant differences (odds ratio) are represented by different letters (ANOVA, GLM, Logit-link, α ≤ 0.05, Tukey). Open circles show outliers above 1.5 times the inter-quartile range.

Finally, *E. formosa* responses to white, green, and yellow backgrounds installed in the LED-enhanced trap indicated that recapture was 54% higher with green and white backgrounds than with the yellow background used for whitefly monitoring. There was also a statistically significant difference in mean response among the three trap types (2.41 ± 0.06, *p* = 0.001) (Figure 7).

## 4. Discussion

Trapping insect pests on sticky cards in greenhouses is a common technique for monitoring within integrated pest management (IPM). Sticky cards, typically yellow or blue and coated on both sides with adhesive, attract and capture flying insects. These traps provide valuable information on pest population status, including abundance, development, distribution, seasonality, and weather dynamics [7]. In addition to pest monitoring, sticky cards can also be used to monitor beneficial insects important for biological control. By regularly examining trap catches, growers can track both pests and natural enemies to support informed management decisions [6]. Yellow sticky cards are generally preferred because they attract a broad range of pests, including fungus gnats, whiteflies, aphids, and psyllids [7,25,26,27], whereas blue cards are used for thrips due to their specific attraction to this color [8].

Both the greenhouse whitefly, *Trialeurodes vaporariorum*, and its parasitoid, *Encarsia formosa*, are attracted to yellow sticky traps, with parasitoid attraction particularly pronounced when host resources are depleted [6,28]. *Trialeurodes vaporariorum* shows heightened attraction to green LED traps [11], consistent with our multiple-choice test in which *E. formosa* also strongly preferred green LEDs. Stukenberg [19] further showed strong whitefly attraction to a green LED-enhanced yellow trap; however, in our experiments, *E. formosa* did not respond to this trap, with or without hosts, indicating a divergent visual response between parasitoid and host. Similar findings were reported by Chen et al. [17] and Chu et al. [18], although mechanisms were not discussed, while McCormack [29] reported the opposite, i.e., attraction of *E. formosa* to a green LED-enhanced yellow trap. Differences in trap design may explain these discrepancies: the trap used by McCormack [29] had a larger visible yellow area relative to the LED-illuminated area, whereas our device integrated the yellow background and green LEDs within a single housing, creating a specific mixture of hue and intensity. We hypothesize that the broad spectral reflectance of the yellow card interfered with the narrowband green LED emission, producing a less attractive stimulus via chromatic opponency. Chromatic opponency arises from antagonistic interactions among photoreceptor channels; in *T. vaporariorum* a blue–green opponent mechanism reduces responses to green stimuli in the presence of blue light [11], and blue/UV LEDs can induce dispersal and disturb whiteflies even during foraging [30,31]. Recent work also shows that blue LED illumination of crops can contribute to push–pull and mass-trapping strategies [31]. Further experiments are needed to test opponency involving green and yellow background cues for *E. formosa*. Our results indicate that green LED-enhanced yellow traps can be used to monitor whiteflies without disrupting *E. formosa* activity: the trap was unattractive to the parasitoid in both the presence and absence of potential hosts. In practice, combining such traps with *E. formosa* releases could improve whitefly management by allowing the parasitoid to parasitize whitefly nymphs while traps target alate adult whiteflies, at least until parasitoids migrate at higher densities [6]. Accordingly, LED-enhanced yellow traps may also serve as selective tools for mass trapping whiteflies, especially in combination with additional approaches to disturb pests on the crop [30,31] and selective killing by laser applications, i.e., in the context of advanced push–pull strategies [32].

To test interference between the green LED and yellow background, we replaced the yellow background with white or green backgrounds. Both modifications significantly increased *E. formosa* recapture relative to the yellow-background LED trap, supporting the opponency hypothesis. Trap attributes such as background, edge length, shape, position, and surface texture can influence trapping efficiency [33]. Our findings suggest that delivering “pure” green light without spectral contamination from the background is critical for attracting *E. formosa*. Spectral measurements showed a peak wavelength of 525.06 nm for the yellow-background trap and 524.03 nm for both modified traps; while insects can be sensitive to small wavelength differences, chromatic opponency likely plays a larger role [11,34,35]. Chen et al. [36] also reported *E. formosa* responses to green light near 504 nm. We further found that *E. formosa* was more strongly affected by LED intensity than whiteflies: parasitoid attraction was high up to about 20 μmol m^−2^ s^−1^ for modified green LED traps, whereas *T. vaporariorum* was similarly attracted in this range and showed higher attraction at intensities up to 25 μmol m^−2^ s^−1^. Based on our observations, *E. formosa* tended to migrate to the modified LED trap only after approximately 3 h on host-infested leaves, presumably after host feeding and parasitizing suitable nymphs [15]. This lack of excessive attraction makes traps without a yellow background suitable for monitoring *E. formosa* establishment against predefined thresholds [6]. Because both *E. formosa* and *T. vaporariorum* were attracted to intensities lower than 20 μmol m^−2^ s^−1^, modified traps may enable simultaneous monitoring of the pest and its natural enemy, facilitating decision making in biocontrol. For example, decreasing catches of whiteflies on the LED traps together with increasing catches of *E. formosa* would indicate successful biocontrol [37]. Nevertheless, validation of this hypothesis in practice is necessary.

## 5. Conclusions

In conclusion, LED-enhanced traps offer advantages over conventional yellow sticky cards: they provide narrow-band emissions with tunable intensity and wavelength to match target photoreceptors [11,38], generate little heat [39], emit steadily regardless of ambient light, and have long lifespans [29]. A potential drawback is the attraction of certain natural enemies, potentially disrupting biocontrol; timing LED activation to avoid the peak activity of beneficials may mitigate this. Trap visibility may also be reduced under bright sunlight, which can reach ~100,000 lx at the Earth’s surface [9], diminishing contrast with artificial light. Moreover, trapping insects on sticky surfaces can negatively affect proper species identification, highlighting the need for automated species identification by AI. Nevertheless, implementing LED-enhanced monitoring can be challenging where power supply is limited.

## Figures and Tables

**Table 1:** Specification of the LEDs used.

LED Color	Manufacturer	Type	Peak Wavelength Measured (nm)	Initial Photo Flux (μmol m^−2^ s^−1^)	Adjusted Photo Flux (μmol m^−2^ s^−1^)
UV	Roithner ^1^	H2A1-H365-E	365	14.1	8.1
blue	Osram ^2^	Oslon SSL 80 LB CP7P	466	43.7	8.1
cyan	Roithner ^1^	B5-433-B505	507	24.9	8.1
green	Osram ^2^	B5-433-B525	523	32.1	8.1
yellow	Roithner ^1^	LED 565-O3U	590	8.1	8.1
red	Osram ^2^	Oslon SSL LR CP7P	634	33.8	8.1

^1^ Vienna, Austria; ^2^ Munich, Germany.

## Data Availability

The data presented in this study are openly available in the LUH Data Repository at https://doi.org/10.25835/jq3q3ttc, reference number [40].
